# Association between erectile dysfunction and cardiovascular risk in individuals with type-2 diabetes without overt cardiovascular disease

**DOI:** 10.4103/0973-3930.57345

**Published:** 2009

**Authors:** Babu Lal Meena, Dhanpat Kumar Kochar, Tulsi Das Agarwal, Raghvendra Choudhary, Abhishek Kochar

**Affiliations:** Department of Medicine, S.P. Medical College, Bikaner, Rajasthan, India

**Keywords:** Cardiovascular risk, erectile dysfunction, periscope

## Abstract

**Background:**

Erectile dysfunction in type-2 diabetes may be an independent marker for coronary artery disease. Present study was undertaken to investigate whether type-2 diabetic patients with erectile dysfunction without having overt cardiovascular disease had increased cardiovascular risk.

**Aim:**

To find out correlation between ED and cardiovascular risk in diabetic patients.

**Methods:**

Fifty type-2 diabetic patients were assessed for erectile dysfunction using international index of erectile dysfunction (IIEF-5), which include questionnaire and cardiovascular risk assessment by multiparameter cardiovascular analysis device (periscope).

**Results:**

The prevalence of erectile dysfunction in type-2 diabetics was very high (78%), mild, moderate and severe ED was present in 6, 36 and 36%, respectively. The total cardiovascular risk was more in patients with ED in comparison to patients without ED (34.87 ± 18.82 vs 20.91 ± 11.03 p = 0.002). The mean 10-years coronary risk and cardiac risk was 12.00 + 9.60 and 22.23 + 14.14 (p = 0.029) and 13.36 ± 1.22 and 28.85 ± 4.13 (p 0.002) in patients without ED and with ED respectively. The mean vascular and atherosclerosis risk was 28.73 ± 13.94 and 39.38 ± 19.51 (p > 0.05) and 26.18 ± 10.31 and 33.92 ± 13.40 (p > 0.05) in patients without ED and with ED, respectively. Total cardiovascular risk was found to increase with age, duration of diabetes and HbA1c levels.

**Conclusion:**

The total cardiovascular risk increases with increasing severity of erectile dysfunction in type-2 diabetic patients without having overt cardiovascular disease.

## Introduction

Prevalence of coronary artery disease in type-2 daibetic population is 55% as against 2 - 4% in the general population of comparable age group.[1] Seventy five percent of patients of type-2 diabetes and about 35% of those with type 1 diabetes die from cardiovascular cause.[2]

The coronary artery disease is more prevalent in type-2 diabetics, it is also more severe and they more often have multi vessels disease as compared to age-matched non diabetics.[3]

Another common complication of diabetes is erectile dysfunction with an estimated prevalence of 20-85% (ranging from mild to complete ED) which occurs at an earlier age than in non diabetic men.[4]

ED is defined as inability to achieve and maintain an erection sufficient to permit satisfactory sexual intercourse.[5]

In the Massachusetts male ageing study, men with treated diabetes had a 28% age adjusted prevalence of complete ED (no erection), almost three times higher than the prevalence of complete ED observed in the entire sample of men (10%). It also showed the extremely deleterious epidemiologic link between coronary artery disease, diabetes and ED.[6]

In 2001 Cohn *et al*, advocated the measurement of arterial compliance to identify patients at risk for cardiovascular events before disease becomes apparent. Pulse countour analysis is a newly developed noninvasive method that allows for easy measurement of arterial elasticity.[7] The validity and reproducibility of brachial ankle pulse wave velocity (baPWV) measurement is considerably high and this method seems to be an acceptable marker, reflecting vascular damage.[8]

## Materials and Methods

The study was carried out in 50 type-2 diabetic patients. Diabetes mellitus was diagnosed according to ADA revised criteria.

### Exclusion criteria

1. History of pelvic trauma
2. History of pelvic surgery
3. History of psychiatric disease
4. Men with debilitating disease
5. Men with unfavorable penile anatomy for sexual act

### Patient examination

All the participants were subjected to a detailed history and complete physical examination. Patients were evaluated for presence of vascular (micro and macrovascular) complications, that is, coronary artery disease, cardiovascular disease, peripheral vascular disease, retinopathy, nephropathy and neuropathy. History regarding age, socioeconomic status, personal and family history of diabetes, history of smoking and use of drugs for any ailment, for example, anti-hypertensive drugs, (β blockers) occupational history, history of usual physical activity, duration of diabetes, treatment history of diabetes (i.e. diet and exercise, insulin therapy, oral hypoglycemic agent only, oral hypoglycemic agent in combination with insulin) was taken. Patients were also asked about complaints with their duration. Patients were thoroughly investigated by routine investigations, HbA_1c_ lipid profile, ECG etc.

Assessment of cardiovascular risk was done by multiparameter cardiovascular analysis device (periscope). Periscope is a multi parameter comprehensive and noninvasive cardiovascular analysis device. It is a tool designed to encompass the total chain of atherosclerosis, diagnosis and treatment ranging from early detection and prevention.

Periscope uses the principles of pulse wave analysis and polymechanocardiography.

### Erectile dysfunction

1. The ED is organic or psychogenic determined by
2. Stamp test
3. Testicular sensation test
4. Interruption of urination
5. ED assessment by international index of ED that include questionnaire.[9]

## Result

Table 1 shows the baseline characteristics in the study group. The mean age of patients with ED was significantly high (p<0.001). The mean duration of diabetes in patients with ED was significantly longer (p<0.001). The mean BMI and WHR were not significantly different in the two groups. The mean systolic blood pressure was significantly high in the ED group (p=0.018). The mean fasting blood glucose was found to be significantly higher in patients with ED as compared to those without ED (p=0.046). The mean HbA1c was also significantly higher in patients with ED as compared to those without ED (p=0.004).The mean 10 years coronary risk was significantly higher in the ED group [p=0.029). The mean total cardiovascular risk was significant in the ED group (p=0.002).The mean cardiac risk was also significantly higher in the ED group (p=0.001).The mean vascular risk was found to be higher but statistically insignificant in the ED group (p>0.05), so was the mean atherosclerosis (p>0.05).The mean lipid values (TG, LDL, HDL-C) significantly higher in patients with ED as compared to those without ED.

**Table 1:** Anthropometric, biochemical parameters and cardiovascular risk in study group with and without erectile dysfunction

Characteristic	ED		
	
	Mean ± SD		*P*
		
	Present	Absent (11)	
Age(yrs)	53.28 ± 6.52	44.09 ± 6.83	<0.001
Duration of diabetes	8.94 ± 6.39	2.84 ± 1.99	0.003
BMI	26.12 ± 4.86	24.87 ± 2.87	0.423
WHR	0.94 ± 0.03	0.92 ± 0.03	0.053
Systolic BP	142.23 ± 18.03	131.27 ± 10.48	0.018
Diastolic BP	86 ± 9.83	86.73 ± 9.39	0.828
S. creatinine	1.20 ± 0.55	1.09 ± 0.62	0.586
FBS	153.69 ± 46.52	123.18 ± 30.52	0.046
HbA1c	8.53 ± 1.79	6.66 ± 1.78	0.004
10 years coronary risk	22.23 ± 14.14	12 ± 9.60	0.029
Total cardiovascular risk	34.87 ± 18.82	20.91 ± 11.03	0.002
Cardiac risk	28.85 ± 25.13	13.36 ± 12.22	0.001
Vascular risk	39.38 ± 19.51	28.73 ± 13.94	0.098
Atherosclerosis risk	33.92 ± 13.40	26.18 ± 10.31	0.083
TC	186.26 ± 38.88	158.36 ± 15.83	0.025
LDL	119.33 ± 31.94	97.06 ± 16.98	0.032
HDL	44.03 ± 8.13	50.18 ± 7.05	0.027
VLDL	23 ± 7.17	21.6 ± 4.16	0.541
TG	116.33 ± 37.30	104.09 ± 17.68	0.299

Table 2 shows complications in diabetic patients with and without erectile dysfunction in the study group. Obesity was present in 20% without ED and 80% with ED.Retinopathy was significantly present in only 1 patient without ED and 13 patients with ED. Significant neuropathy was present in all 25 patients with ED. The frequency of nephropathy in patients with ED as compared to those without ED was not significantly different. Hypertension was also significantly more in patients with ED as compared to these without ED.

**Table 2:** Various complications in diabetic patients with and without erectile dysfunction in the study group

Characteristics		Erectile dysfunction			%	χ^2^	*P*
					
		Absent (n = 11)	%	Present (n = 39)			
Obesity	+ (n = 30)	6	20	24	80	0.175	0.676 NS
	− (n = 20)	5	25	15	75		
Retinopathy	+ (n = 14)	1	7.14	13	92.86	4.196	<0.05
	− (n = 36)	13	36.11	23	63.89		
Neuropathy	+ (n = 25)	0	-	25	100	14.103	<0.001
	− (n = 25)	11	44	14	56		
Nephropathy	+ (n = 12)	1	9.09	11	90.91	1.71	0.190 NS
	− (n = 38)	10	26.32	28	73.68		
Hypertension	+ (n = 27)	5	18.52	22	81.48	5.024	0.025
	− (n = 23)	6	26.09	17	73.91		

Analysis of data presented in Table 3 shows that prevalence of ED but not the severity of ED is increasing with age. There was significant correlation between total cardiovascular risk and HbA1c levels [Table 4]. It was also found that total cardiovascular risk increases as the severity of ED increases [Table 5].

**Table 3:** Prevalence of erectile dysfunction in various age groups.

Age	Total	Severity of erectile dysfunction							
		
		Present		Mild		Moderate		Severe	
					
		No.	%	No.	%	No.	%	No.	%
<40	5	1	20	0	-	0	-	1	100
41 - 50	15	11	73.33	3	27.27	5	45.45	3	27.27
51 - 60	25	22	88	0	-	11	50	11	50
>60	5	5	100	0	-	2	40	3	60
Total	50	39		3	6.0	18	36	18	36

**Table 4:** Correlation between total cardiovascular risk and HbA_1c_ levels

HbA_1c_	Total	Total cardiovascular risk					
		
		<20		21 - 40		>40	
				
		No.	%	No.	%	No.	%
<7	14	7	50	4	28.6	3	21.4
7.1 - 8	9	1	11.1	3	33.3	5	55.6
8.1 - 9	13	2	15.4	5	38.5	6	46.2
>9	14	5	35.7	6	42.9	3	21.4
Total	50	15	30	18	36	17	34

**Table 5:** Correlation between ED and total cardiovascular risk

Severity of erectile dysfunction	Total	Total cardiovascular risk					
		
		<20		21 - 40		>40	
				
		No.	%	No.	%	No.	%
Absent	11	5	45.5	5	45.5	1	9.1
Mild	3	1	33.3	1	33.3	1	33.3
Moderate	18	5	27.8	7	38.9	6	33.3
Severe	18	4	22.2	5	27.8	9	50
Total	50	15	30	18	36	17	34

## Discussion

### Prevalence and severity of erectile dysfunction in diabetic patients:

In the present study, out of 50 diabetic male patients, the erectile dysfunction was present in 39(78%) patients. Out of which, 3(6%) had mild ED and 18(36%) patients had moderate and severe ED in each group, respectively.

Various other workers have studied and reported the prevalence of ED in diabetic patients with variable results.

Schiavi *et al*,[10] studied 40 diabetic men, free from other illness or drugs that could affect sexual capacity and 40 age-matched healthy control subjects. ED was present in 77% of patients. Sundaram *et al*,[11] reported that in diabetic patients, the prevalence of ED was 66%. Ledda *et al*,[12] reported that ED was very common among diabetic patients. They had ED at an earlier age and prevalence was 75%. Sassayam *et al*,[13] studied 6112 Japanese male patients from 447 outpatient clinics and found that 81% had some degree of ED. Kloner[14] observed that the prevalence of ED in diabetic patients was about 75%. Sasaki *et al*,[15] reported prevalence of 90% in 1118 male diabetic patients. Prevalence rate was double than that of nondiabetic individuals.

### Effect of age on the erectile dysfunction

In our study, it was observed that prevalence of erectile dysfunction increased with the increase in age. Prevalence increased from 20% in age group of <40 to 100% in age group of >60 years.

Most of the earlier studies had also reported significant correlation between ED and age. The effect of age on prevalence and severity of disease might be due to age-related changes occurring in body and also various other complications that may coexist in diabetic patients, but ultimately the accelerated atherosclerosis is the common denominator for increased prevalence of ED and cardiovascular disease in ageing population.[6,16–18]

### Erectile dysfunction and total cardiovascular risk and various risk factors

In our study, it was observed that total cardiovascular risk was less in patients without ED. Moreover, as severity of ED increased, total cardiovascular risk also increased. Various other workers have reported the correlation between ED, cardiovascular risk and other risk factors such as HT, dyslipidemias and obesity.[13]

In 2002, Jackson *et al*, concluded that erectile dysfunction and cardiovascular disease share severalrisk factors that are similar and commonly coexist. ED in asymptomatic man may be a marker for underlying coronary artery disease.[19]

In 2003, Roth *et al*, studied 1412 Israeli men and found that ED and cardiovascular disease share common risk factors and may be aggravated by medical treatment for reducing them. They concluded that ED is common among patients who are at high risk for cardiovascular disease because of diabetes and or hypertension.[20]

In 2003, Mota *et al*, studied 310 male diabetic patients and observed that ED showed a positive correlation with obesity, stroke, arteriopathy, retinopathy and smoking, but it was not correlated to type of diabetes mellitus, duration of diabetes <10 years, hypertension, IHD, nephropathy, dyslipidemia and alcohol consumption.[21]

In our study, it was also observed that diabetic patients without ED had less than 10-years coronary risk as compared to patients with ED but severity of ED did not correlate significantly with 10-years coronary risk. Various other workers have also reported significant correlation between ED and 10-years coronary risk. Ponholzer *et al*,[22] reported about men participating in a health-screening project in the area of Vienna completed the International Index of Erectile Function - 5 questionnaire (IIEF5) to assess prevalence and severity of ED. The risk for CHD or stroke within 10 years depending on the severity of ED was estimated according to Framingham risk profile algorithms. They concluded that moderate to severe ED, but not mild ED was associated with a considerably increased risk for CHD or stroke within 10 years. These observations are consistent with the result of the present study [Table 6].

**Table 6:** Correlation between erectile dysfunction and 10 years coronary risk

	Total	10 years coronary risk							
		
		<10		11 - 20		21 - 30		>30	
					
		No.	%	No.	%	No.	%	No.	%
Absent	11	6	54.5	2	18.2	3	27.3	0	-
Mild	3	1	33.3	0	-	2	66.7	0	-
Moderate	18	4	22.2	4	22.2	5	27.8	5	27.8
Severe	18	5	27.8	3	16.7	4	22.2	6	33.3

Grover *et al*,[23] studied that prevalence of ED in the primary care setting; importance of risk factors for diabetes and vascular disease. They concluded that cardiovascular disease, diabetes, future coronary risk and increasing fasting glucose levels are independently associated with ED. It remains to be determined if ED precedes the development of these conditions. The results of this study are comparable with those of the above two studies.

Aggressive management of above risk factors will not only improve erectile dysfunction but also prevent development of more serious complications such as CHD and stroke.
